# Contributions of the immune system to the pathophysiology of traumatic brain injury – evidence by intravital microscopy

**DOI:** 10.3389/fncel.2014.00358

**Published:** 2014-11-04

**Authors:** Susanne M. Schwarzmaier, Nikolaus Plesnila

**Affiliations:** ^1^Department of Anesthesiology, University of Munich Medical CenterMunich, Germany; ^2^Institute for Stroke and Dementia Research (ISD), University of Munich Medical CenterMunich, Germany; ^3^Munich Cluster of Systems NeurologyMunich, Germany

**Keywords:** brain trauma, secondary brain damage, inflammation, leukocytes, microglia, innate immune answer, *in vivo* imaging, intravital microscopy

## Abstract

Traumatic brain injury (TBI) results in immediate brain damage that is caused by the mechanical impact and is non-reversible. This initiates a cascade of delayed processes which cause additional—secondary—brain damage. Among these secondary mechanisms, the inflammatory response is believed to play an important role, mediating actions that can have both protective and detrimental effects on the progression of secondary brain damage. Histological data generated extensive information; however, this is only a snapshot of processes that are, in fact, very dynamic. In contrast, *in vivo* microscopy provides detailed insight into the temporal and spatial patterns of cellular dynamics. In this review, we aim to summarize data which was generated by *in vivo* microscopy, specifically investigating the immune response following brain trauma, and its potential effects on secondary brain damage.

## Traumatic brain injury

Traumatic brain injury (TBI) remains one of the major causes of death and severe disability in industrialized countries (Bruns and Hauser, 2003; Tagliaferri et al., 2006). It results in immediate primary damage that is caused by the mechanical impact and is non-reversible. This primary contusion initiates a cascade of secondary processes on a cellular, subcellular, and molecular level which cause additional—secondary—brain damage (Kontos et al., 1981; Baethmann et al., 1988; Allan and Rothwell, 2001; Sahuquillo et al., 2001; Bramlett and Dietrich, 2004; Nortje and Menon, 2004; Werner and Engelhard, 2007; Harhangi et al., 2008; Maas et al., 2008; Greve and Zink, 2009; Shlosberg et al., 2010): Both vasogenic and cytotoxic brain edema, generated by a disruption of the blood brain barrier (BBB) or swelling of astrocytes, respectively, lead to a raise in intracranial pressure (ICP; Unterberg et al., 2004). This results in reduced cerebral blood flow (CBF) and finally ischemia (Bouma et al., 1992; Golding et al., 1999). CBF is also impaired by alterations in the cerebral microcirculation, e.g., microthrombus formation, the generation of leukocyte/platelet aggregates, and the interaction of leukocytes with the cerebral endothelium (Schwarzmaier et al., 2010, 2013). Inflammatory processes result in the production of NO and free radicals, and the release of chemokines and cytokines which worsen BBB disruption and tissue damage and maintain the inflammatory reaction. Further damage is mediated by apoptotic and necrotic processes in neurons, glia cells, and endothelial cells. While the contusion volume, i.e., the area of irreversible neuronal cell death, reaches its peak already in the first 24–48 h as demonstrated in animal models (Kochanek et al., 1995; Zweckberger et al., 2003, 2006; Engel et al., 2008; Turtzo et al., 2014), ongoing processes orchestrating both inflammatory and recovery-related mechanisms may influence functional outcome and recovery over the following days, weeks and months (Kontos et al., 1981; Allan and Rothwell, 2001; Sahuquillo et al., 2001; Bramlett and Dietrich, 2004; Nortje and Menon, 2004; Unterberg et al., 2004; Werner and Engelhard, 2007; Harhangi et al., 2008; Maas et al., 2008; Greve and Zink, 2009; Shlosberg et al., 2010).

Most data on the pathophysiology of secondary brain damage has been generated in various animal models of TBI. The two most frequently used models mimic the two main features of TBI, i.e., cortical contusion (Controlled Cortical Impact, CCI), and diffuse axon damage (fluid percussion injury, FPI). However, so far none of the available models simultaneously mimics all features of TBI—e.g., additional vessel injury or systemic hypoxia (Lighthall et al., 1989; Finnie and Blumbergs, 2002; Morales et al., 2005; Morganti-Kossmann et al., 2010).

## Inflammation

TBI causes tissue damage and, consequently, induces an acute as well as a chronic inflammatory reaction, including the innate and the adaptive immune system. Both protective and detrimental aspects for the progression of secondary brain damage have been associated to different aspects of the immune response, depending e.g., on the (immune) cell type, the intensity of activation, and on the temporal and spatial relation of the immune response in relation to the initial brain injury (Whalen et al., 1999a; Allan and Rothwell, 2001; Morganti-Kossmann et al., 2001, 2002; Konsman et al., 2007; Rivest, 2009; Loane and Byrnes, 2010; Prinz et al., 2011; de Rivero Vaccari et al., 2014; Peruzzotti-Jametti et al., 2014). In this review we will mainly focus on *in vivo* microscopy studies investigating the pathophysiology of TBI. To be able to put the findings after TBI in the right context, we also included some particularly relevant studies on spinal cord injury (SCI) and cerebral ischemia in our review.

## *In vivo* imaging

The main difficulty in determining the effects of inflammatory cells on secondary brain damage following TBI lies in the nature of the employed methods: histological data provide extensive information on spatial distribution of immune cells as well as their state of activation; however, these data will always remain only a snapshot of processes that are, in fact, very dynamic. In contrast, *in vivo* imaging, and more specifically *in vivo* microscopy, provides detailed insight into the temporal and spatial patterns of cellular and sometimes subcellular dynamics in the living brain. The two main *in vivo* imaging techniques used in animal research are epi-fluorescence and multiphoton microscopy (Denk et al., 1990; Helmchen and Denk, 2005; Shaner et al., 2005; Misgeld and Kerschensteiner, 2006; Xu et al., 2007; Holtmaat et al., 2013). For epi-fluorescence microscopy, fluorophores are excited by light and emit a fluorescent signal which is detected by a CCD camera at high speed. Main shortcomings of this technique are phototoxic tissue damage caused by high excitation energy, and the acquisition of only superficial fluorescent signals, thereby allowing only imaging in two dimensions. Multiphoton microscopy overcomes these problems by an elegant method: two—or more—photons sent consecutively by a laser arrive in the focal point of the objective at almost the same time. Their combined energy results in emission of only one photon with higher energy, i.e., longer wavelength. This phenomenon—the 2-photon effect—results in emission of fluorescence only in the focal point of the objective, and in improved tissue penetration. The consequence is a greatly improved signal to noise ratio which allows imaging of photons deriving deep from brain tissue without the necessity to use high intensity excitation energy which may damage the tissue of interest.

In contrast to histological techniques, *in vivo* imaging needs to (a) stain the cells of interest in the living animal, (b) use surgical techniques to expose the area of interest; and (c) keep animals under anesthesia for several hours. These interventions may interfere with the evolving immune response after TBI. For example, leukocytes were mostly imaged following *in vivo* staining, e.g., with Rhodamine 6G (Villringer et al., 1991), while microglia were studied in most cases in mice expressing CX3CR1-GFP (Jung et al., 2000). For superficial imaging, an open cranial window preparation was employed (Wahl et al., 1985). In contrast, for multiphoton microscopy either the skull was thinned (Frostig et al., 1990), or a bone flap and the underlying dura mater were removed and a cover glass was implanted (Levasseur et al., 1975; Kienast et al., 2010); a procedure already activating microglia and influencing dendritic spine turnover (Xu et al., 2007). The maintenance of sufficient anesthesia and of physiological parameters like body temperature, mean arterial blood pressure (MABP), and arteriolar blood gases (i.e., pH, pO_2_ and pCO_2_, electrolytes, etc.) both during surgery and the time of *in vivo* imaging is another important aspect. These parameters can significantly affect (patho-) physiological processes; consequently, their continuous monitoring and maintenance throughout *in vivo* imaging is important. While the body temperature is controlled and maintained in most *in vivo* imaging experiments discussed in this review, only very few studies provide information on MABP or blood gases (Härtl et al., 1997a,b; Utagawa et al., 2008; Schwarzmaier et al., 2010, 2013), or heart rate and oxygen saturation (Masuda et al., 2011). Since experiments including the preparation of a cranial window and/or imaging can last up to several hours—and the animal is under anesthesia for an equally long period of time—it is important that the ventilation of the animals is sufficient and adjustable to the individual animal. However, only few studies report that the animals were intubated (Masuda et al., 2011) or intubated and ventilated (Härtl et al., 1997a,b; Utagawa et al., 2008; Schwarzmaier et al., 2010, 2013; Herz et al., 2011). Accordingly, these points need to be critically taken into consideration when interpreting data obtained by *in vivo* imaging.

## Cerebral inflammatory response—resident cells

Microglia are the resident macrophages in the brain (Stoll and Jander, 1999; Soulet and Rivest, 2008; Ransohoff and Cardona, 2010; Kettenmann et al., 2011). Under physiological conditions, they have a ramified shape with small cell bodies and long processes which continuously scan their environment (Davalos et al., 2005; Nimmerjahn et al., 2005), monitoring synapses and responding to their functional state (Wake et al., 2009). Upon activation, microglia change both functionally and morphologically into their activated forms which are referred to as “M1” or “M2”. M1 is considered to be the pro-inflammatory state, associated with actions such as phagocytosis, the presentation of antigens, and the production of reactive oxygen species (ROS) and NO. By contrast, microglia with the activation state M2, which is sometimes subdivided further into “acquired deactivation” and “alternative activation”, are responsible for effects such as the fine tuning of inflammation, the recruitment of regulatory T-cells, and for scavenging of debris. They are also associated with the promotion of tissue remodeling or repair, and with angiogenesis (Stoll and Jander, 1999; Colton, 2009; Loane and Byrnes, 2010; Ransohoff and Cardona, 2010; David and Kroner, 2011; Kettenmann et al., 2011). Accordingly, microglia are believed to play both a beneficial and a detrimental role after brain injury, depending—among other factors such as age—both on the injury type, and the time investigated, i.e., the acute or the chronic inflammatory response (Block et al., 2007; Glezer et al., 2007; Rivest, 2009; Loane and Byrnes, 2010; Kettenmann et al., 2011; Nayak et al., 2012; Hernandez-Ontiveros et al., 2013; Peruzzotti-Jametti et al., 2014).

**Figure 1:**
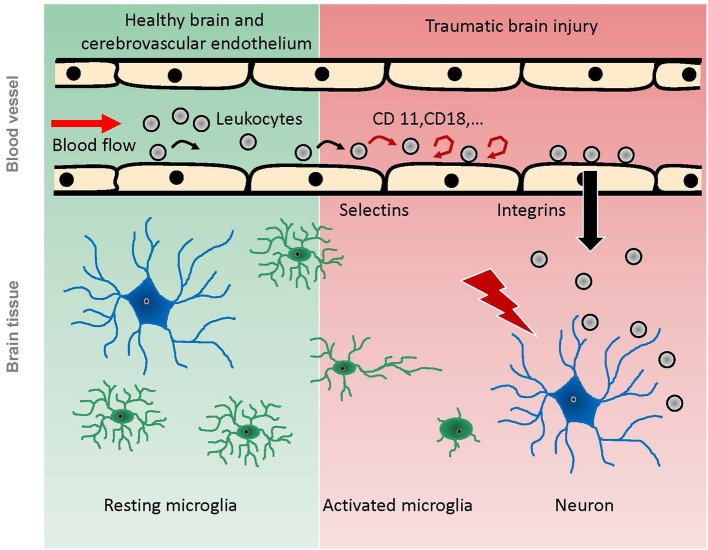
**Scheme of pathophysiological reactions of leukocytes and microglia after traumatic brain injury as demonstrated by *in vivo* experiments**. Under physiological conditions (green background), leukocytes pass the cerebral microcirculation in undisturbed blood flow, while some of them occasionally role on the endothelium. Microglia have a ramified shape and continuously scan the brain parenchyma with their processes. Following TBI (red background), the intravascular leukocytes start rolling and adhering to the endothelium, mediated by selectins and integrins respectively. Finally, they migrate into the damaged tissue. Microglia become activated by brain trauma, extend their processes towards the site of injury, and finally migrate towards the injury, taking up an amoeboid shape.

Following brain injury, microglia extend their processes towards the damaged area as shown *in vivo* after laser or microelectrode injury (Davalos et al., 2005), or *ex vivo* on organotropic hippocampal slice cultures following MCAo (Neumann et al., 2008; Figure 1). Microglia morphologically become more amoeboid and finally migrate towards the site of injury (Kim and Dustin, 2006). These changes in morphology and/or migration resulted in encapsulation of the damaged area (Davalos et al., 2005; Kim and Dustin, 2006), or in engulfment of invading neutrophils (Neumann et al., 2008). The laser injury was performed by high laser power delivered to a dedicated area of interest for a certain time, and the microelectrode injury was induced with a glass electrode which was inserted into the cortex by a micromanipulator (Davalos et al., 2005). Both laser injury and microelectrode injury result in a very small, focal brain damage. Consequently, these techniques provide excellent models for studying very subtle alterations of cells or even subcellular processes in a well-defined area. While these studies generate valuable information on microglia and their functions, they do not mimic clinical brain injury. The dynamics of a TBI, however, can cause a much stronger and more complex damage—depending on injury severity and mechanism—which might affect or activate the resident immune cells quite differently.

Considering that TBI may also lead to cerebral ischemia, the reaction of microglia to ischemic events *in vivo* might also be helpful for a better understanding of the function of microglia after brain trauma. Severely decreased CBF initiated microglial de-ramification—i.e., activation—in different models of cerebral ischemia, while a moderate decrease or an increase in CBF had no visible effect on microglia and their processes (Masuda et al., 2011). Following ischemia, microglia seem to influence the fate of synapses in ischemic areas (Wake et al., 2009). The authors conclude that microglia detect the functional state of synapses and play a role in remodeling neuronal circuits. In both studies several different models of cerebral ischemia were investigated, such as photo-thrombotic stroke and global ischemia. CBF in the region of interest was assessed and directly compared and matched with alterations observed in microglia. Another study on cortical microhemorrhages induced by laser injury showed a local, inflammatory response including activated microglia, however this was limited to an area in close proximity around the lesion (Rosidi et al., 2011).

In contrast to their acute response, the chronic activation of microglia seems to mediate mainly detrimental effects, e.g., via (inadequate) release of cytotoxic chemokines, neurotoxic effects of receptor activation/upregulation (e.g., Toll-like receptors (TLRs)), or ROS production, as reviewed in detail by others (Stoll and Jander, 1999; Block et al., 2007; Rivest, 2009; Loane and Byrnes, 2010; Kettenmann et al., 2011; Giunta et al., 2012; Mannix and Whalen, 2012; Hernandez-Ontiveros et al., 2013).

In data obtained from histological sections *ex vivo*, different properties of acute and chronic activation post trauma were revealed. Microglia displayed mostly a classical activation (M1) or acquired deactivation at seven and up to 28 days after FPI, but no alternative activation (Cao et al., 2012). In line with that, M2 induced by CCI peaked already at day 3–5 post injury (Turtzo et al., 2014). Inhibition of microglia activation 1 month after CCI in mice resulted in less lesion progression at 3 months post injury as assessed by MRI (Byrnes et al., 2012). Unfortunately, so far, little information is available on the chronic activation of microglia following TBI *in vivo*.

## Systemic inflammatory response—blood borne leukocytes

The contributions of the systemic inflammatory response to secondary brain damage following TBI have been investigated intensively (Kochanek and Hallenbeck, 1992; Rothlein, 1997; Ransohoff and Tani, 1998; Johnson-Léger et al., 2000; Ransohoff et al., 2003; Callahan and Ransohoff, 2004; Imhof and Aurrand-Lions, 2004; van Buul and Hordijk, 2004; David and Kroner, 2011). Within the first hours following TBI, leukocytes and leukocyte-platelet aggregates begin to roll on and adhere to the cerebrovascular endothelium (Härtl et al., 1997b; Schwarzmaier et al., 2010, 2013; Figure 1). These studies were performed using clinically relevant trauma models such as FPI and CCI, which mimic not all, but the main features induced by TBI (Lighthall et al., 1989; Finnie and Blumbergs, 2002; Morales et al., 2005; Morganti-Kossmann et al., 2010). Similar leukocyte-endothelium interactions (LEI) have been demonstrated in models of cerebral ischemia (Kataoka et al., 2004) and SAH (Ishikawa et al., 2009). In a model of liver inflammation, LEI was shown to activate cerebral microglia and alter neuronal excitability (D’Mello et al., 2013). For the development of secondary brain damage after TBI, however, rolling and adherence of leukocytes to the cerebral endothelium may have a limited pathophysiological relevance. *In vivo* data showed that BBB breakdown was not associated to LEI following FPI (Härtl et al., 1997a,b), which was confirmed by *ex vivo* data (Whalen et al., 1998, 1999b). Inhibition of leukocyte adherence to the endothelium did not have any effect on secondary lesion progression after CCI (Schwarzmaier et al., 2013). Following laser injury, leukocytes were not recruited into the injury focus but to perivascular spaces in close proximity to the injury as shown *in vivo*; however, this happened not before day one after injury (Kim and Dustin, 2006). There is, however, one study reporting a correlation between leukocyte adherence and vascular leakage 36 h following CCI *in vivo* (Pascual et al., 2013). In this study, however, the craniotomy for the CCI was not resealed, a procedure well known to prevent secondary brain injury after TBI (Zweckberger et al., 2003, 2006). In this setup, post trauma edema formation will not increase ICP and microcirculatory alterations are likely to differ significantly compared to a trauma model where the bone flap is resealed and intracranial hypertension is allowed to build up. These differences of pathophysiological processes with and without intracranial hypertension might well explain the differences in the observed effects of LEI.

While leukocyte rolling and adherence can be monitored by IVM, studying the migration into the affected tissue is technically demanding and has therefore been studied mostly *ex vivo*. Neutrophil depletion has been demonstrated to reduce secondary brain damage, but the effect only became significant at 2 weeks after CCI (Kenne et al., 2012). Another study investigating the effect of an antibody directed against a subunit of the CD11d/CD18 integrin on leukocytes reports a positive effect on lesion volume already 3 days after FPI (Utagawa et al., 2008). In a study conducted by our own laboratory, leukocytes were shown to migrate into the tissue only after secondary brain damage had already occurred (Schwarzmaier et al., 2013), findings in accordance with histological data published by others (Mathew et al., 1994; Holmin et al., 1995; Soares et al., 1995; Holmin and Mathiesen, 1999).

More conclusive *in vivo* data on the role of microglia vs. peripheral immune cells after SCI were presented by Evans et al. (2014). In this study, different chimeras of transgenic mice were generated in order to image either resident microglia or blood borne monocytes/macrophages and their respective contribution to the progression of the injury *in vivo*. The authors show that it is in fact blood-derived macrophages which facilitate secondary axon dieback, and not resident microglia. Invasion of blood borne monocytes/macrophages into the CNS was dependent on microglial TLR4 (Zhou et al., 2006). Accordingly, microglia do not only directly influence secondary brain damage, but also indirectly by recruiting blood borne immune cells.

## Adaptive immune response

To date, the adaptive immune response to TBI and its contributions to secondary brain damage has not been investigated intensively. There are, however, reports indicating both a neuroprotective and a detrimental role of the adaptive immune system in the pathophysiology following SCI or in chronic neuronal diseases, which have been reviewed in detail by others (Ankeny and Popovich, 2009; Schwartz et al., 2013; Rodrigues et al., 2014; Walsh et al., 2014). Histological data suggest that adaptive immune cells migrate into the damaged tissue only after secondary brain damage has occurred (Holmin et al., 1995, 1998; Soares et al., 1995; Schwarzmaier et al., 2013).

One study investigating the behavior of T-cells in health and autoimmunity *in vivo* showed that naïve T-cells did not migrate into the healthy CNS, while they partly migrated into inflamed brain tissue after Experimental Autoimmune Encephalomyelitis (EAE) *in vivo*. In their paper, the authors show that the migratory capacity of T-cells depends more on the activation status rather than the phenotype or antigen specificity (Herz et al., 2011).

## Summary/outlook

TBI induces an inflammatory reaction which includes both the innate and the adaptive immune system. This inflammatory response can be roughly divided into an acute phase lasting hours to days, and a chronic phase lasting for weeks to months or even years. Upon TBI, microglia shift to different forms of activation, which have both detrimental and beneficial effects on secondary brain damage. By and large, the acute phase of microglia activation seems to provide various protective and beneficial mechanisms, reaching from phagocytosis to recovery and repair, while a chronic response is mainly associated with negative effects on the CNS. Following focal brain injury, data obtained *in vivo* demonstrate that microglia extend their processes towards the damaged area, change morphologically into a more amoeboid shape, and finally migrate towards the site of injury. This can result in the encapsulation of the damaged area or in the engulfment of invading leukocytes. While these observations were reported following laser injury or microelectrode injury, *in vivo* experiments on microglia activation in a clinically more relevant TBI model such as CCI or FPI are still missing. Additionally, no *in vivo* data is available on chronic microglia activation.

The systemic part of the inflammatory response following TBI has been studied intensively. It includes blood borne leukocytes which interact with the cerebrovascular endothelium and finally migrate into the damaged tissue. While this might influence outcome in other diseases, such as the early stage after a stroke, it does not seem to be of great importance for the mechanisms involved in secondary contusion expansion following TBI. However, a chronic response could worsen functional outcome and recovery.

Therapeutic alterations of the innate immune system might be promising not only in models of chronic inflammation, but also in TBI, specifically in view of the different forms of microglia activation after acute brain injury. Further studies are needed in order to investigate therapeutic options targeting inflammation, and to fully elucidate microglia activation *in vivo* following clinically relevant models of TBI.

## Author contributions

Susanne M. Schwarzmaier: Review of the literature and writing the manuscript. Nikolaus Plesnila: Review of the literature and revision of the manuscript. Both authors approve the final version of the manuscript and are accountable for all aspects of the work.

## Conflict of interest statement

The Guest Associate/Review Editor Arthur Liesz declares that, despite being affiliated to the same institution as the authors, the review process was handled objectively and no conflict of interest exists. The authors declare that the research was conducted in the absence of any commercial or financial relationships that could be construed as a potential conflict of interest.
